# Credit System

**Published:** 1854-01

**Authors:** 


*Credit System.*—The credit system, so prevalent in this country, falls with peculiarly oppressive weight upon young professional men, and especially upon young physicians. Many estimable representatives of this latter class spend their all in acquiring medical knowledge, and, upon entering the profession, while they are compelled to pay cash for their board, office rent, and other necessary expenses, are bestowing gratuitous service on those capable of remunerating them, or waiting a year for more honest competent patients to discharge their dues. Consequently many sink under a load of debt, and recruit the ranks of cab-drivers and California miners, or are only saved from this fate by the helping hand of friends.

The members of the Burlington Medical Society have recently resolved that after January 1st, 1854, they will present their bills for professional services at the conclusion of each case of sickness.

The editor of the *N. J. Medical Reporter*, in noticing and advocating the general adoption of this important reform, makes the following judicious remarks:

"The present credit system is objectionable, because the services rendered are personal services; and as the obligation of the physician to the patient ceases with the termination of the illness, the obligation of the patient to the physician should be discharged at the same time. * * *

"The merchant, in disposing of a bill of goods on time, say six or twelve months, calculates that the purchaser will sell at a profit, in order to get the money to pay the creditor, and remunerate himself. Merchandize is the seal of the purchase — and it is often within reach of the seller, even when out of his possession, in the event of the buyer's default. We have no such security. The *commodity* (if such a term is allowable) rendered by us is *advice*, and as it is not, like the merchant's goods, to be bartered or sold again for a profit, we are entitled to its estimated pecuniary value at the time of its bestowal."

We need hardly say how fully we agree in the foregoing observations. We trust the profession will take the matter into consideration.—*Virginia Med. and Surg. Journal.*

